# Supplementary material for: Repetition-related reductions in neural activity support improved behavior through increases in oscillatory power
Source: bioRxiv. 2025 Jul 6:2025.07.06.663291. Preprint. [Version 1] doi: 10.1101/2025.07.06.663291 (PMC12236478; doi:10.1101/2025.07.06.663291)
Supplement: 1 [file NIHPP2025.07.06.663291V1-supplement-1.pdf]

bioRxiv preprint doi: https://doi.org/10.1101/2025.07.06.663291; this version posted July 6, 2025. The copyright holder for this preprint (which was not certified by peer review) is the author/funder. This article is a US Government work. It is not subject to copyright under 17 USC 105 and is also made available for use under a CC0 license.

SUPPLEMENTARY FIGURES AND TABLES

bioRxiv preprint doi: https://doi.org/10.1101/2025.07.06.663291; this version posted July 6, 2025. The copyright holder for this preprint (which was not certified by peer review) is the author/funder. This article is a US Government work. It is not subject to copyright under 17 USC 105 and is also made available for use under a CC0 license.

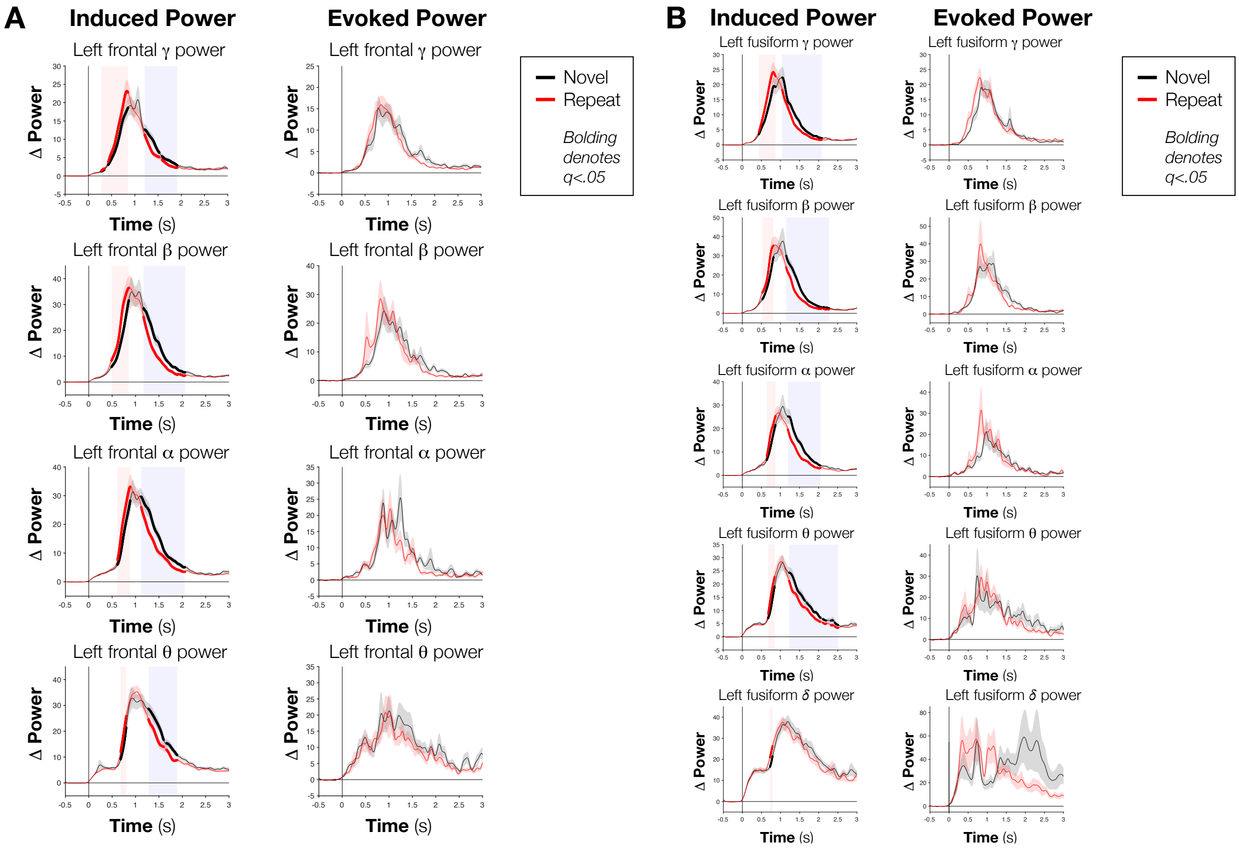

Supplementary Fig. 1. Effects of repeated object identification on induced versus evoked power for the fMRI-defined A) left frontal and B) left fusiform regions. Evoked power corresponds to power estimates after first averaging across trials in the time domain per condition, whereas induced power is calculated per trial after first subtracting the trial-averaged evoked response and then averaging across trials. FDR-corrected periods are shown with bolded lines, with red lines representing change in power relative to the baseline period for the Repeat condition and black lines representing the Novel condition.

bioRxiv preprint doi: https://doi.org/10.1101/2025.07.06.663291; this version posted July 6, 2025. The copyright holder for this preprint (which was not certified by peer review) is the author/funder. This article is a US Government work. It is not subject to copyright under 17 USC 105 and is also made available for use under a CC0 license.

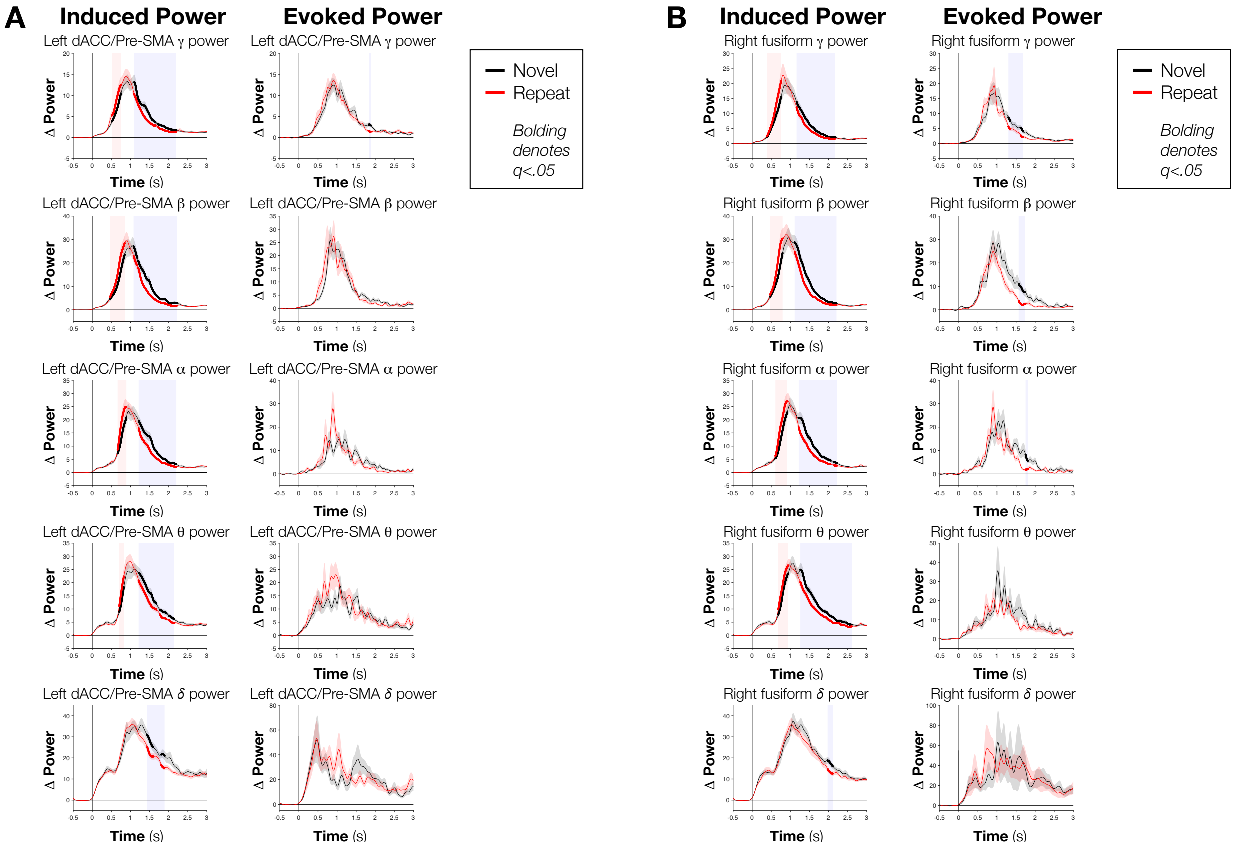

Supplementary Fig. 2. Effects of repeated object identification on induced versus evoked power for the fMRI-defined A) dorsal anterior cingulate/pre-supplementary motor area (dACC/Pre-SMA) and B) right fusiform regions. Evoked power corresponds to power estimates after first averaging across trials in the time domain per condition, whereas induced power is calculated per trial after first subtracting the trial-averaged evoked response and then averaging across trials. FDR-corrected periods are shown with bolded lines, with red lines representing change in power relative to the baseline period for the Repeat condition and black lines representing the Novel condition.

bioRxiv preprint doi: https://doi.org/10.1101/2025.07.06.663291; this version posted July 6, 2025. The copyright holder for this preprint (which was not certified by peer review) is the author/funder. This article is a US Government work. It is not subject to copyright under 17 USC 105 and is also made available for use under a CC0 license.

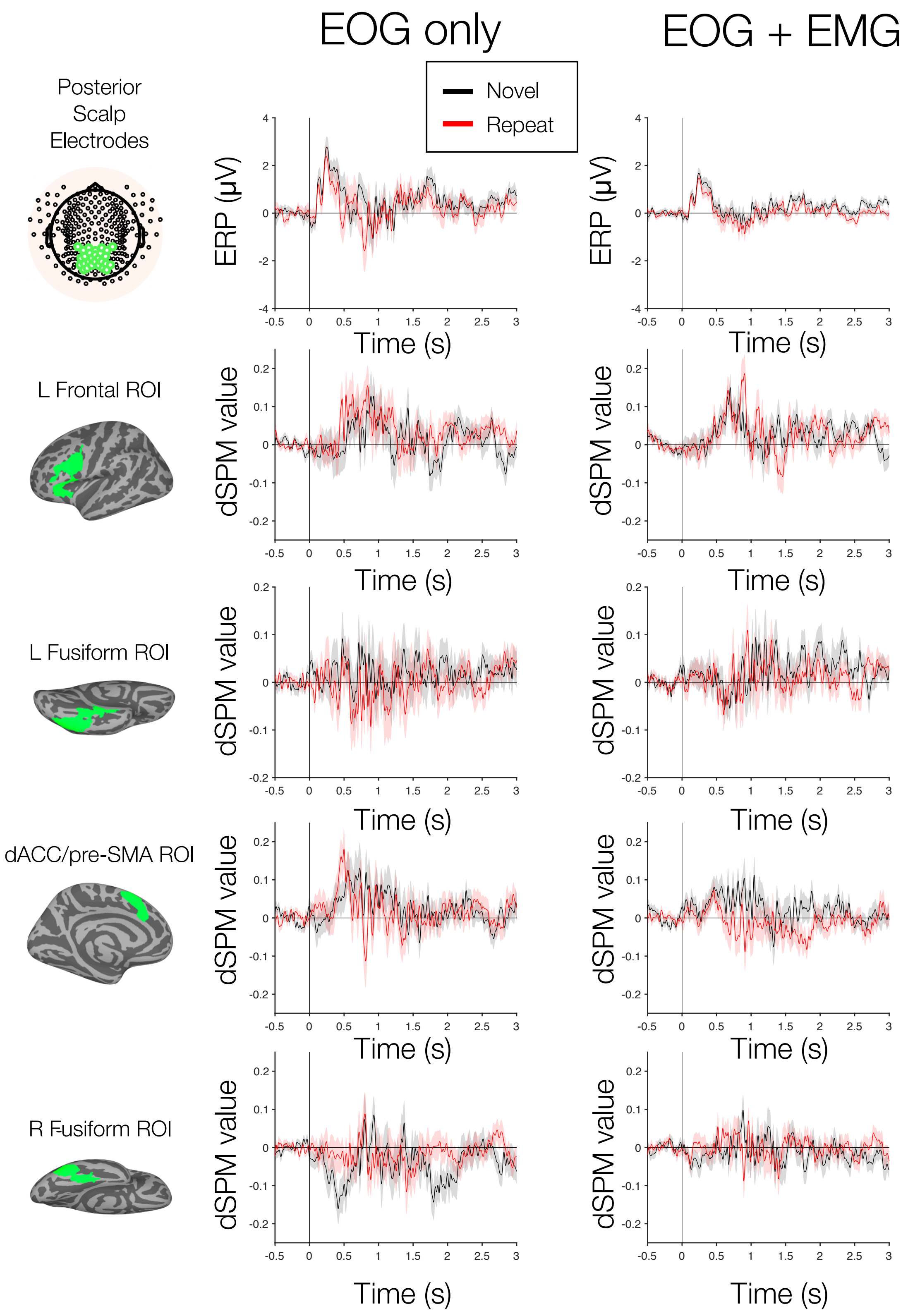

871

872 **Supplementary Fig. 3.** Event related potentials for EOG only (pipeline 1) versus EOG plus
873 speech EMG pre-processing (pipeline 2). Novel and Repeat condition ERPs (black and red lines,

bioRxiv preprint doi: https://doi.org/10.1101/2025.07.06.663291; this version posted July 6, 2025. The copyright holder for this preprint (which was not certified by peer review) is the author/funder. This article is a US Government work. It is not subject to copyright under 17 USC 105 and is also made available for use under a CC0 license.

respectively) are shown in the top row for the selection of posterior scalp electrodes (graphic on left), then for the source estimated time series of the fMRI-defined regions (left frontal, left fusiform, left dACC/pre-SMA, and right fusiform regions). The amplitudes of the ERPs are reduced near the time of the average response time (~ .9 seconds) and .5 seconds before for the EOG+EMG pre-processing relative to the EOG only pre-processing. The estimated ERPs are unitless for the fMRI-defined regions due to the normalization by noise covariance periods.

bioRxiv preprint doi: https://doi.org/10.1101/2025.07.06.663291; this version posted July 6, 2025. The copyright holder for this preprint (which was not certified by peer review) is the author/funder. This article is a US Government work. It is not subject to copyright under 17 USC 105 and is also made available for use under a CC0 license.

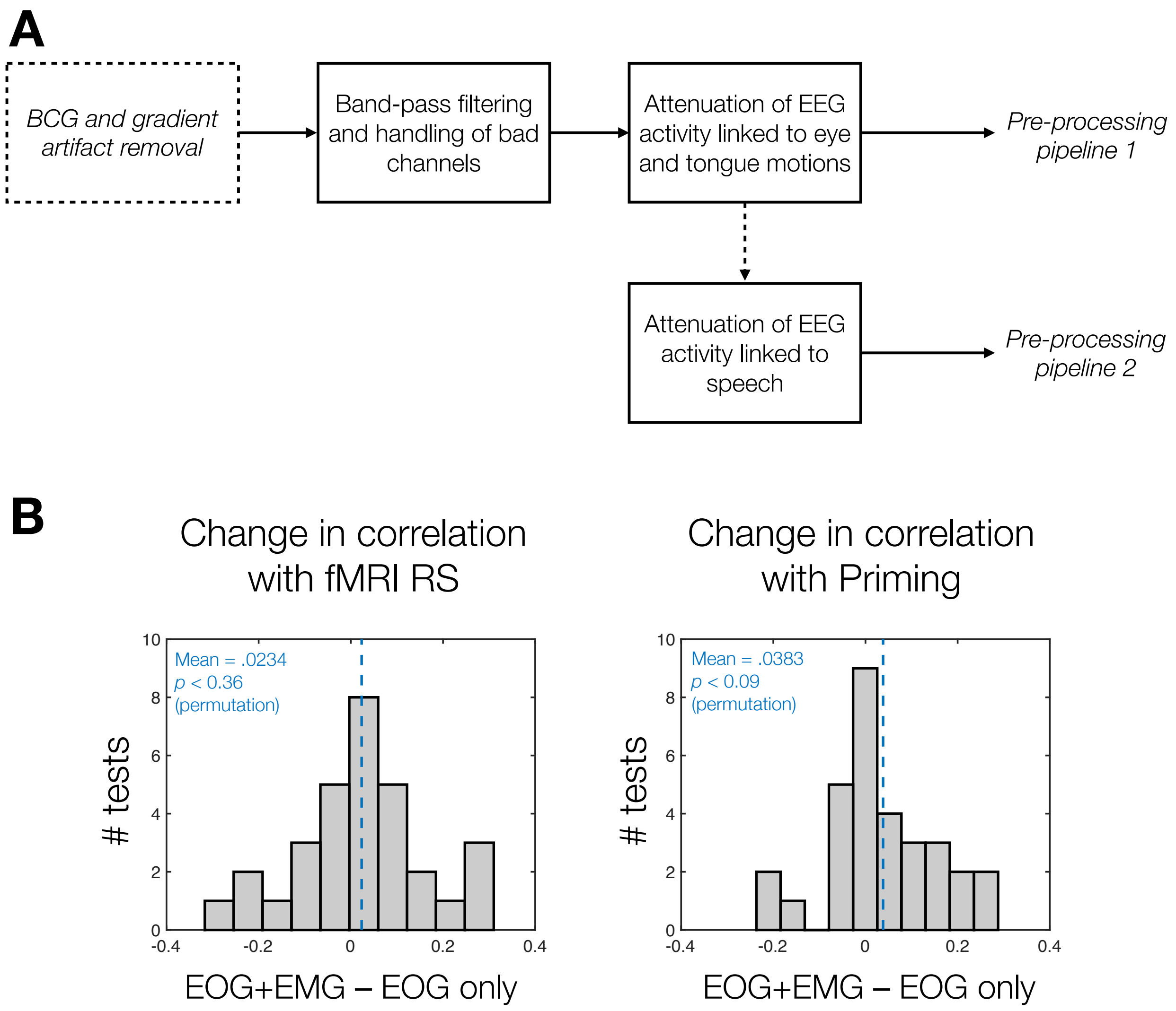

Supplementary Fig. 4. Schematic of different pre-processing pipeline and how these impact correlations of induced power differences (Repeat - Novel) for the early and late periods with fMRI RS and priming. A) Pipeline 1 ("EOG only") regressed signals from electrodes near the eyes and upper jaw to remove EOG and tongue EMG signals, whereas Pipeline 2 ("EOG+EMG") additionally removed signals from the scalp electrodes based on ICAs calculated near the time of the speech response (-0.2 to +0.2 s relative to the RT). See Materials and Methods: EEG Preprocessing for further details. B) Correlations of induced power differences (Repeat - Novel) for the early and late periods with fMRI RS and priming show little change or slightly larger correlations in the EOG+EMG preprocessing (pipeline 2) relative to the EOG only preprocessing (pipeline 1). Shown are frequency histograms of the change in correlation between pipelines across all tests, with mean change values near zero for correlations with fMRI RS and marginally positive for correlations with priming ($p < .09$, permutation test). Negative correlations were first rectified to positive values using absolute value for the purposes of these calculations (e.g. positive values in the histogram reflect a stronger correlation – either originally positive or negative – in the EOG+EMG relative to the EOG only preprocessing). Permutation

bioRxiv preprint doi: https://doi.org/10.1101/2025.07.06.663291; this version posted July 6, 2025. The copyright holder for this preprint (which was not certified by peer review) is the author/funder. This article is a US Government work. It is not subject to copyright under 17 USC 105 and is also made available for use under a CC0 license.

testing (10,000 iterations) was conducted by randomly flipping the signs of the correlation differences and recalculating the mean differences.

bioRxiv preprint doi: https://doi.org/10.1101/2025.07.06.663291; this version posted July 6, 2025. The copyright holder for this preprint (which was not certified by peer review) is the author/funder. This article is a US Government work. It is not subject to copyright under 17 USC 105 and is also made available for use under a CC0 license.

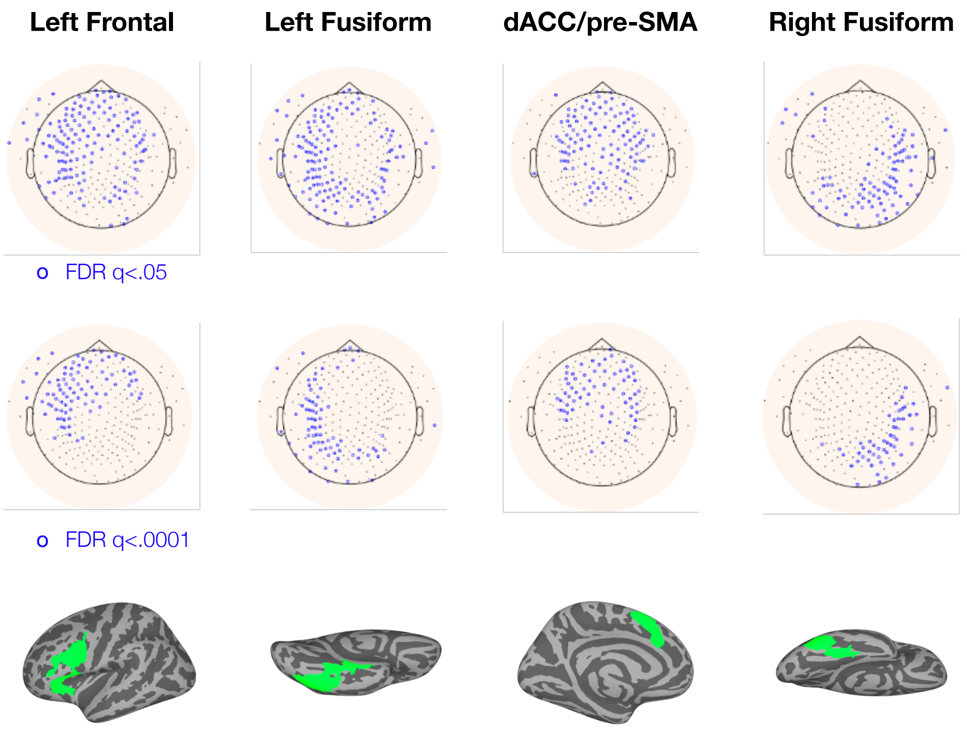

Supplementary Fig. 5. Mapping source regions to their most heavily weighted scalp electrodes. Shown are two FDR-corrected significance levels: FDR $q < .05$ (minimum thresholding for significance) and $q < .0001$ (more stringent thresholding). Some degree of spatial selectivity is observed under both thresholds, although even at the more stringent threshold ($q < .0001$) there is overlap of the identified scalp electrodes across regions – highlighting the more limited spatial resolution of EEG. Electrodes were identified by taking the electrode weights associated with each source vertex in the source estimation process (see Materials and Methods), normalizing them by the standard deviation across those weights, and then finding the mean weight with each surface electrode for all the vertices belonging to a given fMRI-defined region. One-sample *t*-tests across participants were then carried out for each scalp electrode per fMRI-defined region. Multiple comparisons were corrected by FDR. ERPs and induced power differences for these electrode selections are shown in Supplementary Figs. 6-9. While induced power differences appear similar across frequencies and fMRI-defined regions, the ERPs for these electrode selections have distinctive shapes, consistent with a moderate degree of spatial selectivity of the scalp EEG signals.

bioRxiv preprint doi: https://doi.org/10.1101/2025.07.06.663291; this version posted July 6, 2025. The copyright holder for this preprint (which was not certified by peer review) is the author/funder. This article is a US Government work. It is not subject to copyright under 17 USC 105 and is also made available for use under a CC0 license.

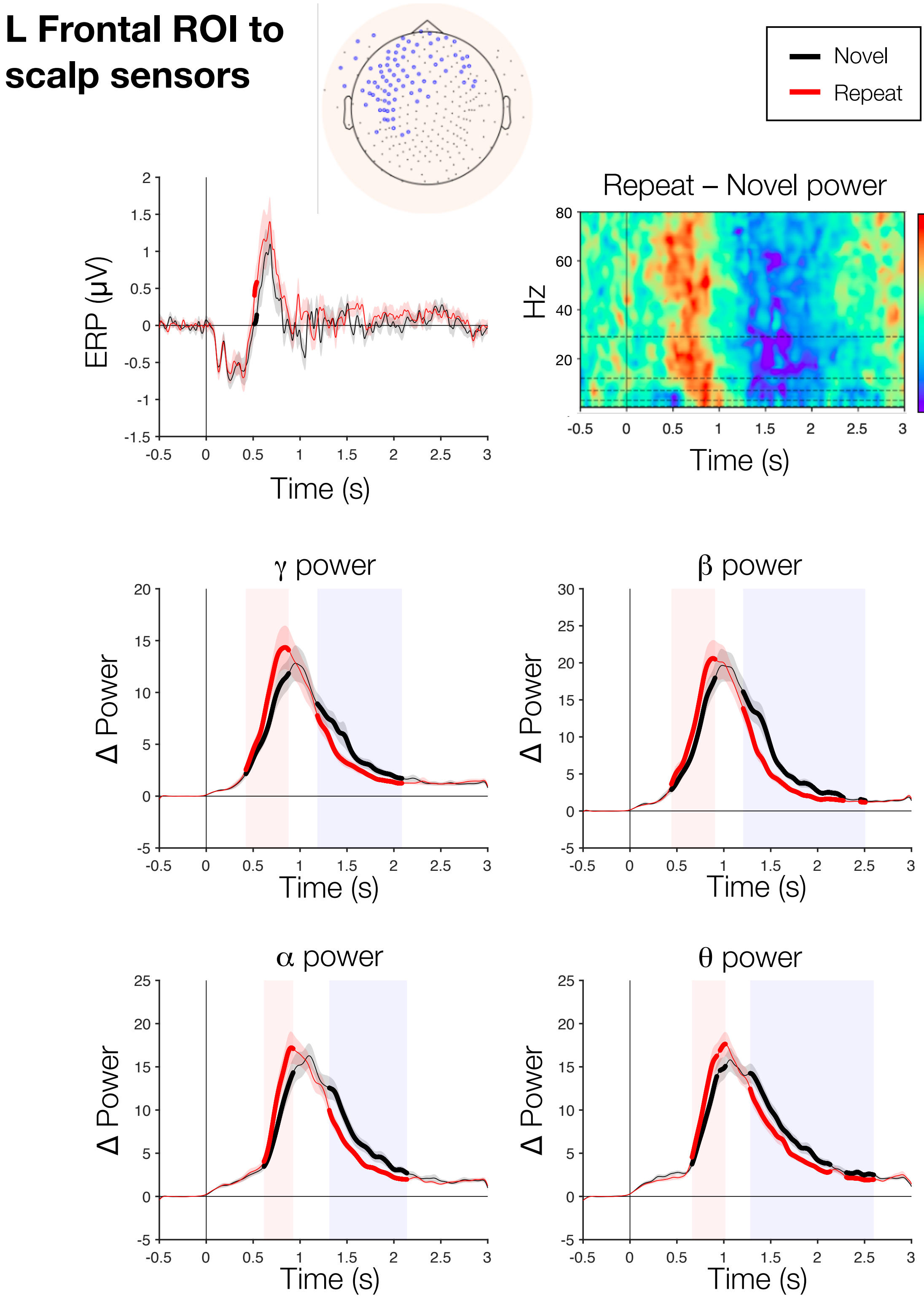

Supplementary Fig. 6. Companion scalp electrode analyses for the fMRI-defined left frontal region (FDR $q < .0001$ from Supplementary Fig. 5). Average ERPs for the defined collection of electrodes (see inset at top) for the Novel (black) and Repeat (red) conditions. Repeat-Novel time

bioRxiv preprint doi: https://doi.org/10.1101/2025.07.06.663291; this version posted July 6, 2025. The copyright holder for this preprint (which was not certified by peer review) is the author/funder. This article is a US Government work. It is not subject to copyright under 17 USC 105 and is also made available for use under a CC0 license.

by frequency induced power differences are shown in the upper right (paired *t*-tests across participants), highlighting the same “early” (Repeat > Novel) and “late” (Novel > Repeat) periods as in the source-estimated results in Fig. 2. Banded power analyses (Novel vs. Repeat) are then shown below for the gamma (30-80 Hz), beta (13-29 Hz), alpha (8-12 Hz) and theta (4-7 Hz) frequency bands, with FDR-corrected periods of significance indicated with bolded lines. While no FDR-corrected periods were detected in the ERPs, robust induced power differences are observed across frequencies.

bioRxiv preprint doi: https://doi.org/10.1101/2025.07.06.663291; this version posted July 6, 2025. The copyright holder for this preprint (which was not certified by peer review) is the author/funder. This article is a US Government work. It is not subject to copyright under 17 USC 105 and is also made available for use under a CC0 license.

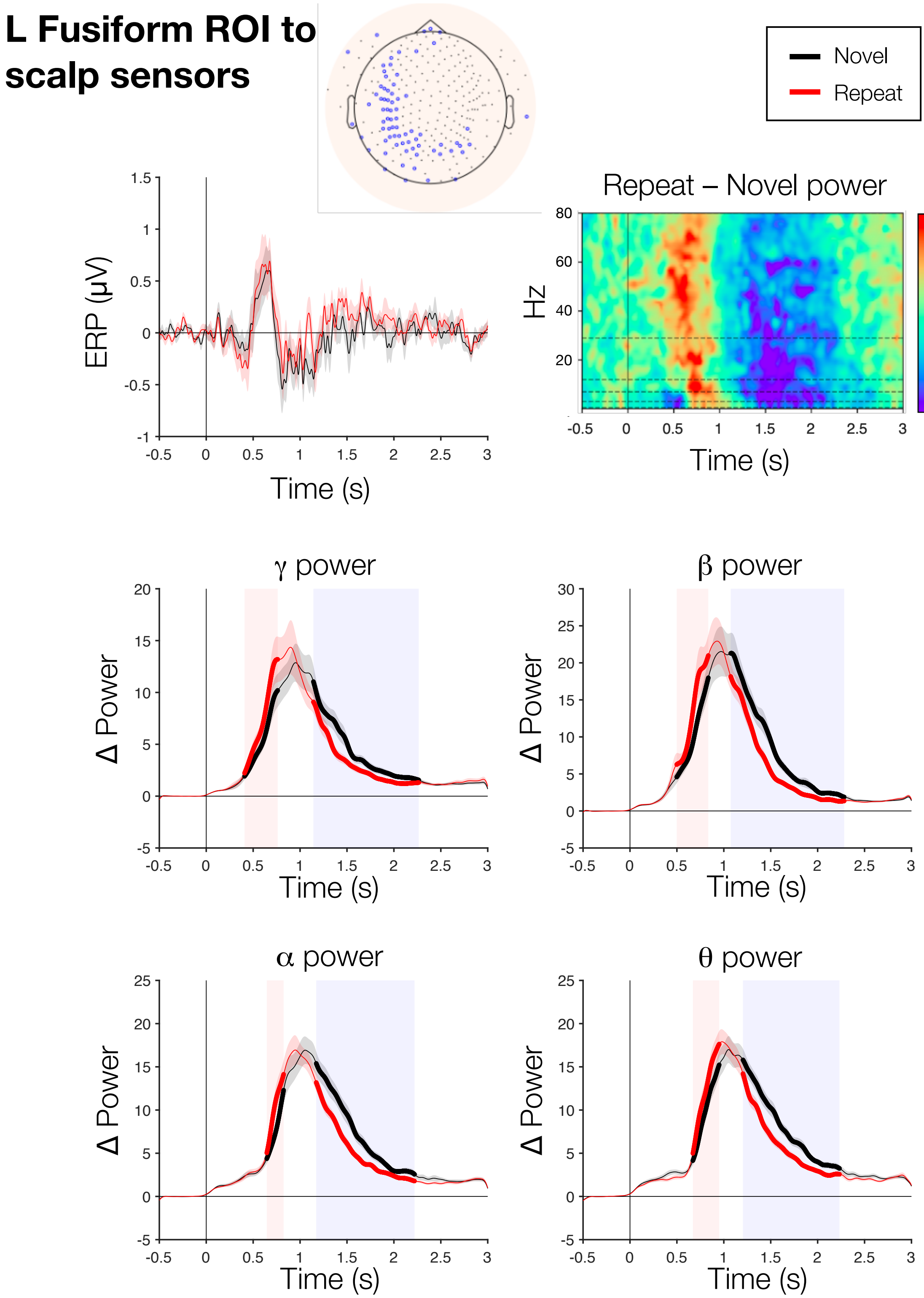

Supplementary Fig. 7. Companion scalp electrode analyses for the fMRI-defined left fusiform region (FDR $q < .0001$ from Supplementary Fig. 5). Average ERPs for the defined collection of electrodes (see inset at top) for the Novel (black) and Repeat (red) conditions. Repeat-Novel time

bioRxiv preprint doi: https://doi.org/10.1101/2025.07.06.663291; this version posted July 6, 2025. The copyright holder for this preprint (which was not certified by peer review) is the author/funder. This article is a US Government work. It is not subject to copyright under 17 USC 105 and is also made available for use under a CC0 license.

by frequency induced power differences are shown in the upper right (paired *t*-tests across participants), highlighting the same "early" (Repeat>Novel) and "late" (Novel>Repeat) periods as in the source-estimated results in Fig. 2. Banded power analyses (Novel vs Repeat) are then shown below for the gamma (30-80 Hz), beta (13-29 Hz), alpha (8-12 Hz) and theta (4-7 Hz) frequency bands, with FDR-corrected periods of significance indicated with bolded lines. While no FDR-corrected periods were detected in the ERPs, robust induced power differences are observed across frequencies.

bioRxiv preprint doi: https://doi.org/10.1101/2025.07.06.663291; this version posted July 6, 2025. The copyright holder for this preprint (which was not certified by peer review) is the author/funder. This article is a US Government work. It is not subject to copyright under 17 USC 105 and is also made available for use under a CC0 license.

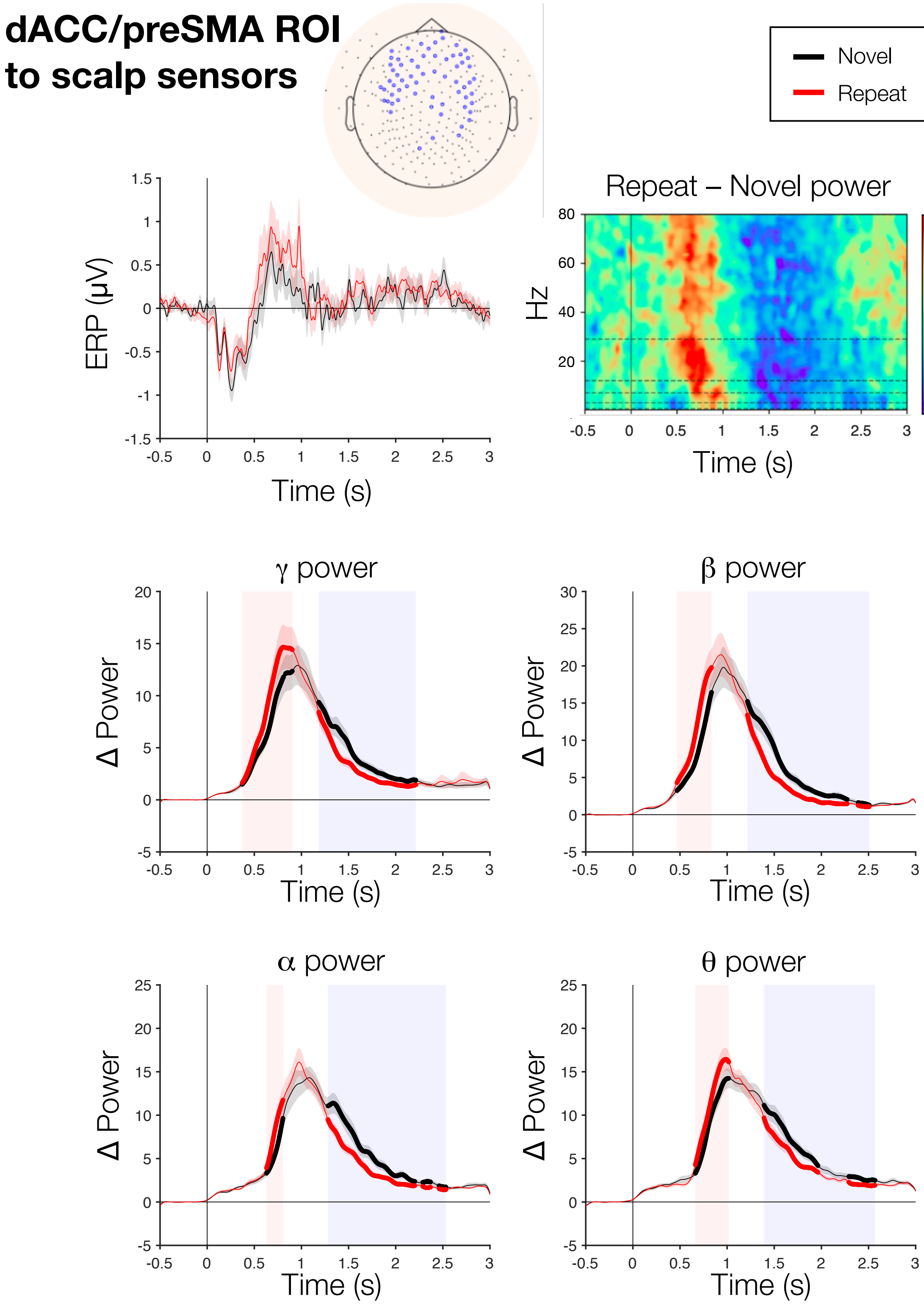

942

943 **Supplementary Fig. 8.** Companion scalp electrode analyses for the fMRI-defined left
944 dACC/pre-SMA region (FDR $q < .0001$ from Supplementary Fig. 5). Average ERPs for the
945 defined collection of electrodes (see inset at top) for the Novel (black) and Repeat (red)

bioRxiv preprint doi: https://doi.org/10.1101/2025.07.06.663291; this version posted July 6, 2025. The copyright holder for this preprint (which was not certified by peer review) is the author/funder. This article is a US Government work. It is not subject to copyright under 17 USC 105 and is also made available for use under a CC0 license.

conditions. Repeat-Novel time by frequency induced power differences are shown in the upper right (paired *t*-tests across participants), highlighting the same “early” (Repeat>Novel) and “late” (Novel>Repeat) periods as in the source-estimated results in Fig. 2. Banded power analyses (Novel vs Repeat) are then shown below for the gamma (30-80 Hz), beta (13-29 Hz), alpha (8-12 Hz) and theta (4-7 Hz) frequency bands, with FDR-corrected periods of significance indicated with bolded lines. While no FDR-corrected periods were detected in the ERPs, robust induced power differences are observed across frequencies.

bioRxiv preprint doi: https://doi.org/10.1101/2025.07.06.663291; this version posted July 6, 2025. The copyright holder for this preprint (which was not certified by peer review) is the author/funder. This article is a US Government work. It is not subject to copyright under 17 USC 105 and is also made available for use under a CC0 license.

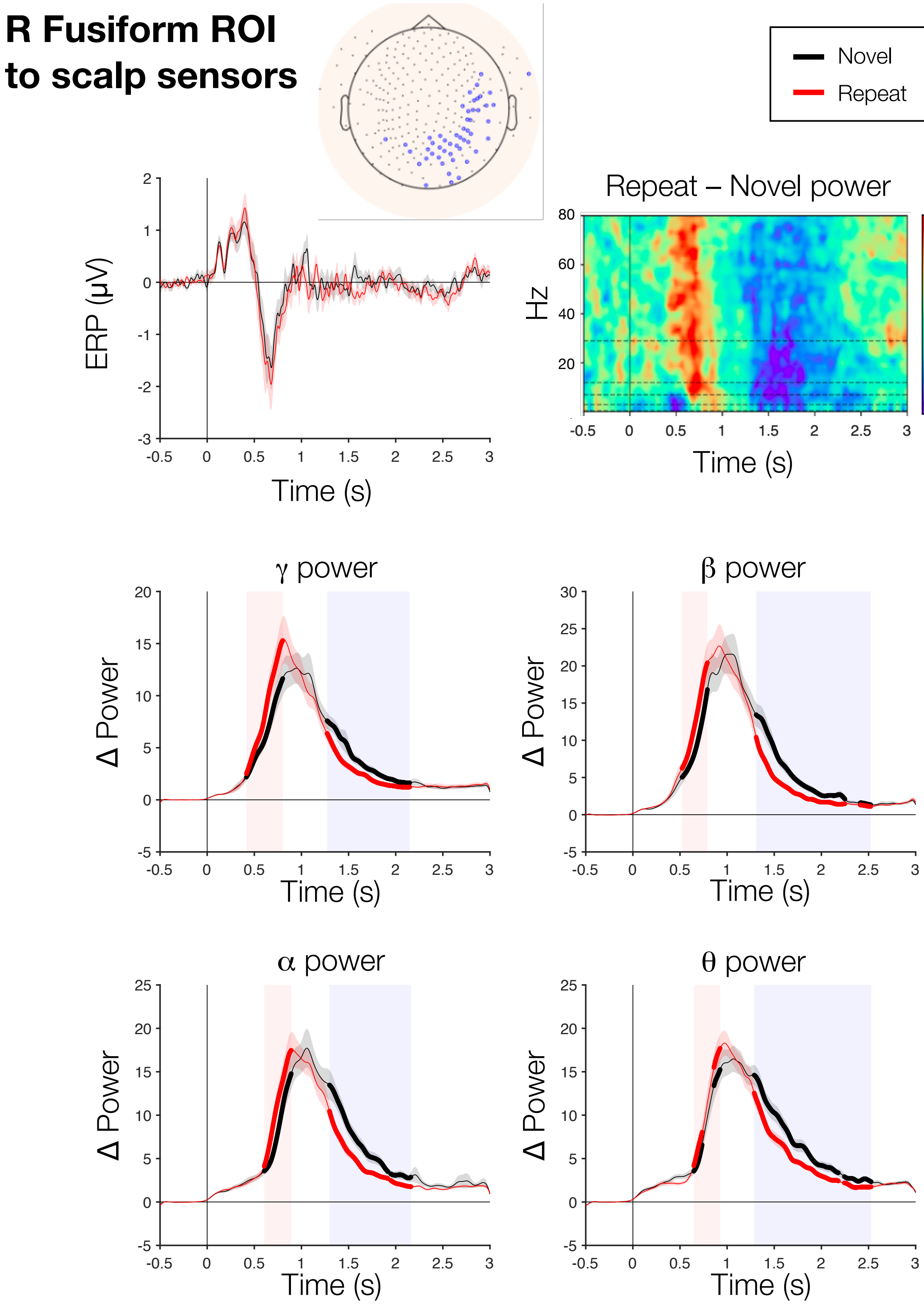

Supplementary Fig. 9. Companion scalp electrode analyses for the fMRI-defined right fusiform region (FDR q <.0001 from Supplementary Fig. 5). Average ERPs for the defined collection of electrodes (see inset at top) for the Novel (black) and Repeat (red) conditions. Repeat-Novel time

bioRxiv preprint doi: https://doi.org/10.1101/2025.07.06.663291; this version posted July 6, 2025. The copyright holder for this preprint (which was not certified by peer review) is the author/funder. This article is a US Government work. It is not subject to copyright under 17 USC 105 and is also made available for use under a CC0 license.

by frequency induced power differences are shown in the upper right (paired *t*-tests across participants), highlighting the same “early” (Repeat>Novel) and “late” (Novel>Repeat) periods as in the source-estimated results in Fig. 2. Banded power analyses (Novel vs Repeat) are then shown below for the gamma (30-80 Hz), beta (13-29 Hz), alpha (8-12 Hz) and theta (4-7 Hz) frequency bands, with FDR-corrected periods of significance indicated with bolded lines. While no FDR-corrected periods were detected in the ERPs, robust induced power differences are observed across frequencies.

bioRxiv preprint doi: https://doi.org/10.1101/2025.07.06.663291; this version posted July 6, 2025. The copyright holder for this preprint (which was not certified by peer review) is the author/funder. This article is a US Government work. It is not subject to copyright under 17 USC 105 and is also made available for use under a CC0 license.

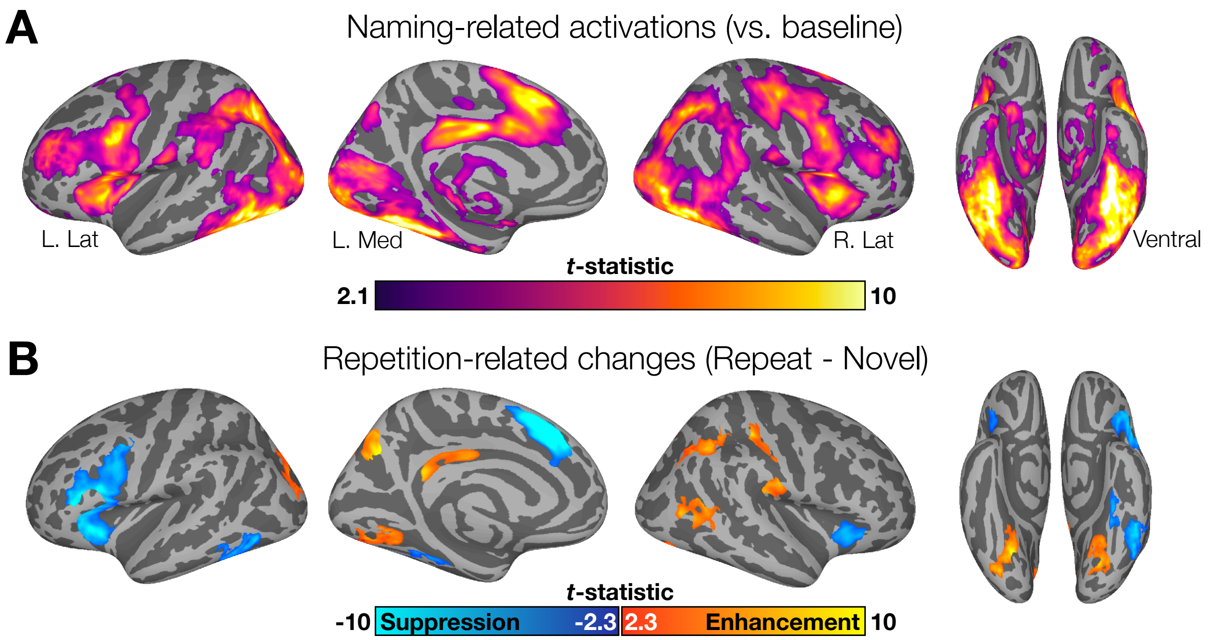

Supplementary Fig. 10. Vertexwise results of Naming-related activations and Repetition effects in the covert naming experiment. (A) fMRI identified significant activations in multiple regions across the cortex, consistent with those observed in the overt naming task. (B) Within Naming-activated regions, significant repetition suppression was noted in frontal and fusiform cortex and were generally left-lateralized. Effects are somewhat attenuated when compared to those in the Overt data, potentially due a combination of the covert nature of the task and the smaller sample size. For display purposes, the map of repetition effects is set to $q < .05$, inclusively masked by regions identified in the earlier Naming-related activation map, while maintaining the same cluster size minimum.
